# Ultra-processed food consumption across early life: implications for pediatric health and disease risk

**DOI:** 10.3389/fnut.2026.1806903

**Published:** 2026-06-11

**Authors:** Maria Elena Capra, Valentina Donini, Martina Muzi, Simone Bruni, Susanna Esposito, Giacomo Biasucci

**Affiliations:** 1 Pediatrics and Neonatology Unit, Guglielmo da Saliceto Hospital, Piacenza, Italy; 2 Department of Medicine and Surgery, University of Parma, Parma, Italy; 3 Pediatric Clinic, University Hospital of Parma, Parma, Italy

**Keywords:** childhood obesity, life-course approach, non-communicable diseases, pediatric nutrition, pregnancy, ultra-processed foods

## Abstract

Ultra-processed foods (UPFs) are industrial formulations characterized by high energy density, low nutritional quality, and the extensive use of additives, and their consumption has increased markedly worldwide. In many high-income countries, children and adolescents now derive up to 50–60% of their total daily energy intake from UPFs, raising major public health concerns. This narrative review synthesizes current evidence on UPF consumption across critical life stages, with a particular focus on pregnancy, childhood, and adolescence, and examines its potential implications for short- and long-term health outcomes. Available evidence consistently links high UPF intake in pediatric populations to excess weight gain, metabolic syndrome, and early cardiovascular risk. Additional adverse outcomes include dental caries and a higher prevalence of allergic diseases, such as atopic dermatitis and asthma. Several biological mechanisms may mediate these associations, including impaired satiety regulation, excessive intake of free sugars and saturated fats, disruption of the food matrix, and alterations in gut microbiota composition, immune function, and inflammatory pathways. Emerging research also indicates that exposure to UPFs may begin before birth, as maternal consumption during pregnancy and lactation has been associated with unfavorable offspring outcomes, including altered neurodevelopment, increased adiposity, and immune-related conditions. Familial, socioeconomic, and behavioral factors strongly influence early exposure to UPFs. Modifiable determinants such as breastfeeding duration, parental nutrition literacy, shared family meals, and screen time represent key targets for preventive interventions. Overall, the evidence highlights the urgent need for life-course–oriented nutritional strategies that promote unprocessed and minimally processed foods, reinforce family-based nutrition education, and support healthy dietary patterns from pregnancy through childhood and adolescence to reduce the long-term burden of non-communicable diseases.

## 1 Background

According to the NOVA food classification system, ultra-processed foods (UPFs) are industrial formulations largely composed of refined substances derived from whole foods—such as sugars, oils, fats, and starches—combined with a wide range of additives intended to enhance flavor, texture, appearance, and shelf life. These products contain little or no intact food components and are specifically engineered to be convenient, highly palatable, and ready to consume. As summarized in Table 1, UPFs represent the most extensively processed category within the NOVA system and are progressively displacing traditional home-prepared meals in contemporary dietary patterns (1).

**Table 1:** Classification of foods according to the NOVA system.

NOVA group	Description	Examples	Main characteristics/processing purpose
Group 1: Unprocessed or minimally processed foods	Natural foods altered by minimal processes such as cleaning, removal of inedible parts, freezing, or pasteurization, without added substances.	Fresh fruits and vegetables, grains, legumes, eggs, milk, meat, fish, nuts.	Maintain most of the natural structure and nutrient content; processing aims to preserve or make edible.
Group 2: Processed culinary ingredients	Substances extracted from Group 1 foods or nature, used in culinary preparations.	Oils, butter, sugar, salt, honey, starches.	Usually not consumed alone; used to season, cook, or combine foods.
Group 3: Processed foods	Products manufactured by adding ingredients from Group 2 to Group 1 foods.	Canned vegetables, cheeses, freshly baked breads, cured meats, simple jams.	Processing enhances durability or palatability; food still recognizable as derived from natural sources.
Group 4: Ultra-processed foods (UPFs)	Formulations of ingredients derived from foods and additives, containing little or no intact Group 1 foods.	Soft drinks, packaged snacks, reconstituted meat products, instant soups, breakfast cereals, frozen ready meals.	Industrial formulations engineered for hyper-palatability, long shelf life, and convenience; typically high in sugar, fat, sodium, and low in fiber and micronutrients.

The contribution of UPFs to total dietary energy intake varies substantially across countries but is particularly high in high-income nations. UPFs account for approximately 42% of daily energy intake in Australia and up to 58% in the United States. In contrast, lower consumption levels have been reported in countries such as Italy (approximately 10%) and South Korea (approximately 25%). Although Italy is a high-income country, UPF consumption is lower than expected based on income level, likely reflecting the persistent influence of the Mediterranean diet, which remains deeply embedded in national food culture (2). Supporting this observation, a recent cross-sectional study involving 1,936 Italian adults showed that individuals adhering more closely to healthier dietary patterns—such as the Mediterranean diet, the Dietary Approaches to Stop Hypertension, the Alternate Diet Quality Index, and the Diet Quality Index–International—consumed fewer UPFs and more unprocessed or minimally processed foods. While UPF intake in southern Italy was broadly consistent with that reported in other Mediterranean countries, higher consumption was associated with a poorer nutrient profile, highlighting the need to monitor UPF intake before their increasing availability and popularity further influence the dietary habits of younger generations (3).

In low- and middle-income countries, including Mexico and Colombia, UPFs account for 16–30% of total daily energy intake. Over recent decades, the availability, affordability, and diversity of UPFs have expanded rapidly worldwide, driven by industrialization, urbanization, and globalization of food systems. As a result, in several high-income countries, UPFs now account for a substantial proportion of total daily energy intake among the general population (2, 4, 5).

A growing body of epidemiological evidence links high consumption of certain types of UPFs to adverse health outcomes. An umbrella meta-analysis reported consistent associations between increased intake of UPFs and a higher risk of cardiometabolic diseases, common mental health disorders, and all-cause mortality. Moreover, UPF consumption is frequently associated with sedentary lifestyles, which may further exacerbate health risks (1). In pediatric populations, family and lifestyle factors appear to be particularly influential. Oliveira et al. conducted a population-based cross-sectional study involving parent–child dyads aged 6–11 years and demonstrated that higher UPF intake among children was strongly associated with unhealthy parental eating habits, excessive screen time, and high parental UPF consumption (all *p* < 0.05). These findings underscore the central role of the home food environment in shaping children’s dietary behaviors and support the inclusion of family-based lifestyle interventions in public health policies (6).

From a life-course perspective, early exposure to unhealthy dietary patterns is especially concerning. The Developmental Origins of Health and Disease (DOHaD) framework, also known as the “Barker hypothesis,” proposes that interactions between genetic susceptibility and environmental factors—particularly nutrition—shape the risk of chronic diseases that begin in utero and extend through infancy and childhood. Exposures occurring during the critical “first thousand days,” from conception through early postnatal life, may induce long-lasting metabolic and physiological adaptations. Barker’s seminal work demonstrated that low birth weight and early-life undernutrition are associated with an increased risk of ischemic heart disease in adulthood, suggesting that nutritional deprivation during early development promotes energy-conserving adaptations that predispose individuals to cardiometabolic disorders later in life (7, 8).

Emerging evidence suggests that exposure to UPFs may begin even before birth. Maternal consumption of some UPFs during pregnancy and lactation has been associated with adverse outcomes in offspring, including an increased risk of atopic dermatitis (9) and potential impairments in neuropsychological development (10, 11). These findings indicate that maternal diet quality plays a critical role in shaping early immune and neurodevelopmental trajectories, reinforcing pregnancy as a key window for nutritional prevention. Within this rapidly evolving nutritional landscape, traditional dietary patterns are increasingly being replaced by food environments dominated by ultra-processed products, including some highly processed plant-based alternatives.

Pregnancy, childhood, and adolescence, therefore, represent particularly vulnerable periods. In many countries, children and adolescents derive a substantial proportion of their daily energy intake from UPFs, with frequent consumption of ready-to-eat meals and sweet snacks reflecting a marked shift toward highly processed dietary habits (12, 13). Identifying modifiable determinants of excessive UPF consumption during childhood is essential for promoting healthier dietary patterns and reducing long-term disease risk (14, 15). The main epidemiological studies assessing UPF intake among pediatric populations across different countries are summarized in Table 2.

**Table 2:** Summary of key indicators of UPFs consumption among children and adolescents worldwide.

Country	Study	Population involved	Main indicator (summary)	Reference
Italy	OKkio alla SALUTE—Italian National Institute of Health (ISS)	Primary school, grade III (~46,000 children, nationally representative)	More than 50% consume sweet snacks more than three times per week, and around 25% drink sugar-sweetened beverages daily.	(9)
Italy	Fondazione Aletheia (2025)	National sample of children	Over half of participants reported eating sweet snacks >3 times/week; a notable increase in the consumption of ready-to-eat and ultra-processed foods was observed.	(10)
Italy, Spain, Portugal, Egypt, and Lebanon	DELICIOUS Project (2025)	Children and adolescents (6–17 years) across five Mediterranean countries (including Italy)	High consumption of ‘unhealthy UPF’ was associated with poorer dietary quality; results refer to the 6–17 year multi-country sample.	(78)
Italy	INHES (2010–2013)	National Italian cohort (5–19 years)	Ultra-processed foods accounted for ≈ 25.9% of total energy intake, serving as a historical benchmark for youth dietary patterns.	(24)
Belgium, Cyprus, Estonia, Germany, Hungary, Italy, Spain, and Sweden.	I.Family study.	146 children aged 6–9 years and 645 adolescents aged 10.19 years	UPFs accounted for 48.6% of total daily energy for children and 47.5% for adolescents	(33)
Spain	Childhood Obesity Risk Assessment Longitudinal Study (CORALS)	Preschool children (aged 3–6 years) were recruited from schools and centers in 7 cities in Spain.	33% of the study population consumed 76.7 g/day of energy adjusted UPFs.	(29)
Spain	Eating Healthy and Daily Life Activities study	26 adolescents aged 12–17 years	Average UPFS consume was 1642.5 g per day.	(79)
Brazil	Cross-sectional data from the 2008–2009 Brazilian Dietary Survey	30,243 individuals aged ≥10 years	UPFs represented 30% of the total energy intake.	(80)
Brazil	The Brazilian National Survey on Child Nutrition (ENANI-2019), a household-based population survey	14,558 children <5 years residing in 123 Brazilian municipalities	The prevalence of consumption of at least one UPF group was 81% in Brazilian children <5 years, with the most consumed UPFs being sweet or savory biscuits/cookies (51.0%), sweetened beverages (37.5%), baby cereals (29.4%) and yogurt (28.1%).	(81)
United States of America	Cross-sectional data from the National Health and Nutrition Examination Survey from 2013 to March 2020	10,102 subjects aged 2–19 years	65.4% of energy derived from UPF s and 25.1% from MPFs in the whole sample.	(70)
Greece	Sample from the Hellenic National Nutrition and Health Survey (HNNHS).	443 children aged 2–18 years	The average percentage of total daily energy provided by UPFs was 39.8%	(82)
Kenya	cross-sectional household study among adolescents	621 subjects aged 10–19 years	25.2%of daily energy derived from UPFs	(83)
Lebanon	cross-sectional national survey	893 children aged 6 months to 4.9 years	UPFs were found to contribute 47% of daily Energy intake	(84)
Peru	surveys used multistage stratified random samples. 24-h recalls were applied on random days per participant,	2,887 children between 6 and 35 months of age	UPFs provided an average of 27% of total energy and were consumed by 86%.	(85)
Canada	CHILD Cohort Study	2,217 children aged 3–5 years	UPF accounted for 45.0% of the daily energy intake at age 3. Males contributed more UPF energy than females (46.0% vs. 43.9%; *p* < 0.001).	(71)

From an evolutionary perspective, the profound environmental changes in diet and lifestyle initiated by agriculture and animal domestication approximately 10,000 years ago—and further accelerated during the Industrial Revolution—have occurred too recently for the human genome to fully adapt. This mismatch between ancient genetic programming and modern nutritional environments has been implicated in the rise of so-called “diseases of civilization.” In particular, food-processing methods introduced during the Neolithic and Industrial periods have substantially altered glycemic load, fatty acid composition, macronutrient balance, micronutrient density, and fiber content, potentially contributing to the growing burden of chronic diseases in Western societies (16).

Against this background, the present narrative review aims to provide a comprehensive overview of current evidence on ultra-processed food consumption across key life stages, with a specific focus on pregnant women, infants, children, and adolescents. Specifically, this review examines the potential impact of UPFs on maternal health and fetal development, as well as on growth, metabolic health, immune function, neurodevelopment, and the risk of non-communicable diseases in pediatric populations, highlighting critical windows of vulnerability and opportunities for early, nutrition-based prevention strategies.

## 2 Methods

A comprehensive literature search was conducted using the MEDLINE–PubMed, Scopus, Web of Science, Embase, and Cochrane Library databases to identify relevant publications from 2005 to 2025. The search strategy targeted peer-reviewed studies, including randomized placebo-controlled trials, controlled clinical trials, double-blind randomized controlled trials, and systematic reviews.

The following keyword combinations were applied consistently across all databases: (“ultra-processed” AND “food”) OR (“ultra-processed” AND “foods”) AND (“child” OR “children” OR “pediatric” OR “childhood” OR “adolescence”). No database-level filters were applied during the electronic search. The search was restricted to full-text articles published in English-language journals.

To ensure completeness, a manual search of the reference lists of all eligible articles was also performed. In addition, two independent researchers conducted a targeted hand search of key journals, conference proceedings, and bibliographies to identify potentially relevant studies that may not have been captured through electronic searches. This process involved page-by-page screening and citation tracking to enhance the review’s comprehensiveness, reproducibility, and depth.

Studies were excluded if they were not available in English, were published outside the predefined time frame, or did not fall within the specified study designs. Additional exclusion criteria included the use of food classification systems other than NOVA, the absence of a clear definition or cited reference for UPF exposure, the use of non-UPF dietary exposure variables, and studies focusing exclusively on overall dietary patterns without specific assessment of ultra-processed food consumption.

This work was designed as a selective narrative review, defined as a critical appraisal of the most relevant and methodologically robust literature addressing the impact of ultra-processed food consumption on health outcomes in children and adolescents. Screening of retrieved records was performed independently by two reviewers. In cases of disagreement regarding study eligibility, a third reviewer was consulted to evaluate the record and reach a final consensus.

## 3 Results

### 3.1 Early-life determinants of unhealthy dietary patterns

A wide range of familial, individual, and socioeconomic factors influences children’s eating behaviors, which are often established early in life and tend to persist into adulthood. Determinants of future health can be identified even before birth. According to the Developmental Origins of Health and Disease (DOHaD) theory, also known as the “Barker hypothesis,” lifestyle-related disorders originate during conception and extend through the embryonic, fetal, and neonatal periods as a result of interactions between genetic predisposition and environmental exposures. During the so-called “first thousand days,” from conception onward, both positive and negative influences may exert long-lasting effects on health trajectories (7). Barker originally demonstrated an association between low birth weight, early-life undernutrition, and the development of ischemic heart disease in adulthood, suggesting that nutritional deprivation during early development promotes energy-conserving adaptations that increase susceptibility to cardiometabolic disorders later in life (8).

Consistent with this framework, a recent study in a Czech pregnancy cohort from the early post-communist era examined associations among dietary patterns, maternal characteristics, and birth outcomes. An “unhealthy” dietary pattern—characterized by high consumption of meat, processed foods, and sweets—was associated with lower birth weight and a higher cephalization index after adjustment for confounding variables. In contrast, a “healthy/traditional” dietary pattern, marked by high intakes of whole-meal bread, dairy products, fruits, and vegetables, was not associated with adverse birth outcomes. These findings support current recommendations promoting balanced and nutritious diets during pregnancy (17).

Early environmental exposures also play a key role in shaping behavioral development. Data from the HELIX (Human Early Life Exposome) project, encompassing six European birth cohorts, examined the relationship between a wide range of prenatal and childhood environmental factors and behavioral outcomes in children aged 6–11 years. Among the 88 prenatal and 123 childhood exposures assessed, maternal smoking and driving during pregnancy showed the strongest correlations with adverse outcomes. Conversely, longer sleep duration, adherence to a healthy diet, and greater family social capital were associated with more favorable behavioral profiles, whereas higher exposure to heavy metals, indoor air pollution, and unhealthy dietary patterns were linked to poorer outcomes (18).

Several studies highlight the role of family-related factors in shaping children’s consumption of UPFs. The SENDO study, conducted among 809 Spanish children aged 4–5 years, reported a mean UPF intake of 37.64% of total energy intake. Larger family size was associated with higher UPF consumption, whereas greater parental awareness of children’s eating behaviors was linked to lower intake. Among child-related factors, shorter breastfeeding duration and longer screen time were both associated with higher UPF consumption (14). These findings may partly reflect socioeconomic constraints, as UPFs are often inexpensive, require minimal preparation, and are readily available as ready-to-eat or ready-to-heat products, making them particularly attractive for larger households.

Parental nutrition education and role modeling strongly influence children’s dietary habits. Sharing family meals and consuming the same foods as parents are among the strongest predictors of higher vegetable intake during preschool years (19), as well as reduced frequency of fast-food consumption and UPF intake. In contrast, watching television during meals appears to negate many of the benefits associated with shared family meals (20). Screen exposure during eating may promote overconsumption by diverting attention from food intake and increasing susceptibility to food advertising. Moreover, excessive screen time outside mealtimes has been associated with more frequent consumption of UPF-based snacks (21, 22).

**Figure 1:**
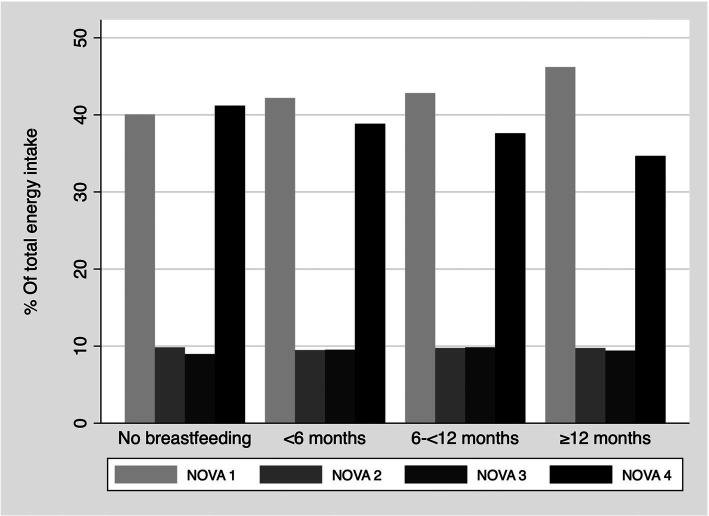
Distribution of total energy intake by NOVA food group according to breastfeeding duration among children aged 4–5 years enrolled in the Child Follow-Up for Optimal Development project (January 2015–June 2021). Reproduced from (25), licensed under CC BY-NC-ND 4.0.

Breastfeeding practices represent another critical early-life determinant. Exclusive breastfeeding for the first 6 months of life is recommended by the World Health Organization. A large Brazilian study involving six-year-old children found that breastfeeding for less than 3 months and introducing complementary foods before 4 months were associated with higher UPF consumption later in childhood (23). These findings were corroborated by additional analyzes from the SENDO study, which demonstrated an inverse linear relationship between breastfeeding duration and UPF intake at 5 years of age. Children breastfed for longer periods consumed more unprocessed or minimally processed foods (NOVA Group 1), such as fruits, vegetables, nuts, and potatoes, whereas those who were never breastfed or breastfed for shorter durations had higher intakes of UPFs (NOVA Group 4) (24) (Figure 1). Overall, evidence consistently indicates that prolonged breastfeeding is associated with lower UPF consumption and a greater preference for nutrient-dense, minimally processed foods later in childhood (25).

### 3.2 UPFs and non-communicable diseases in childhood

#### 3.2.1 Weight excess

UPFs are increasingly replacing minimally processed and traditional foods in the diets of children and adolescents worldwide, raising growing concerns regarding their health implications, given that some UPFs are considered unhealthy. Multiple international studies have documented both the extent and persistence of this phenomenon. For instance, the SENDO study estimated that UPFs contribute approximately 36% of total daily energy intake among Spanish children (14). Similarly, the German KOPS longitudinal study showed that UPF exposure tends to persist and even increase over time, rising from about 24% of total energy intake at age 8 to nearly 27% in young adulthood (26).

A robust body of evidence links higher UPF consumption to poorer overall dietary quality. Diets rich in UPFs are typically characterized by excessive intakes of saturated fats, added sugars, and sodium, coupled with low fiber and micronutrient density, contributing to the development of cardiometabolic disorders (27, 28). In the CORALS study, a baseline cross-sectional analysis of Spanish preschoolers aged 3–6 years, UPF consumption was associated with obesity and other cardiometabolic risk factors, supporting the need for public health strategies that promote the replacement of UPFs with unprocessed or minimally processed foods (29).

Mechanistic insights have been provided by the Ultra-Processed Foods in Obesity (UFO) Project, which examined the role of dietary advanced glycation end-products (AGEs) in pediatric obesity. Compared with healthy controls, children with obesity consumed higher amounts of UPFs, energy, AGEs, and saturated fats, and exhibited increased skin AGE accumulation and mitochondrial dysfunction in peripheral blood mononuclear cells. Similar mitochondrial alterations were observed when healthy cells were experimentally exposed to AGEs, suggesting a causal role of UPF-related compounds in metabolic dysfunction (30).

Modeling studies further support the potential benefits of reducing UPF intake. Livingston et al. developed a microsimulation model using data from the National Health and Nutrition Examination Survey and a randomized controlled trial on UPF reduction. The model predicted that lowering UPF consumption could result in a median BMI reduction of −2.09 kg/m^2^ among U.S. children and adolescents aged 7–18 years, with substantial implications for obesity prevention (31).

UPFs are engineered to be highly palatable yet have poor satiating capacity due to low fiber content and disruption of the natural food matrix, promoting excessive energy intake (32). Data from the Pelotas birth cohort showed that higher UPF intake among children aged 2–4 years was associated with significantly higher BMI-for-age Z-scores. Moreover, the low protein content of UPF-based diets may induce compensatory overeating to meet protein requirements, further contributing to excess energy intake and weight gain (32). Longitudinal evidence from the KOPS study confirmed that adolescents in the highest quartile of UPF consumption were more than twice as likely to be overweight or obese in early adulthood compared with those in the lowest quartile, even after adjustment for confounders (33). However, it is important to note that the overall UPF consumption discussed in this publication may not accurately reflect the varied effects of specific food groups within the UPF category, some of which have beneficial effects.

High UPF consumption is also associated with dyslipidemia, insulin resistance, and hypertension due to elevated intakes of saturated fats, free sugars, and sodium (27, 28, 34). When central obesity, dyslipidemia, hypertension, and insulin resistance cluster, they define metabolic syndrome (MetS). Several studies, including Brazilian adolescent cohorts, have reported a positive association between UPF intake and MetS prevalence (34), consistent with findings from a recent systematic review (28). Although the European I.Family study did not find a significant association between UPF intake and MetS prevalence overall, children with the highest UPF consumption were overrepresented among those with the most adverse metabolic profiles (33).

Collectively, these findings indicate that sustained UPF consumption during childhood is a major risk factor for long-term cardiometabolic disease. While physical activity remains essential for maintaining healthy weight, excessive UPF intake may attenuate its protective effects (34). Thus, UPFs may represent both a cause and a consequence of pediatric overweight and obesity. A limitation of many of these studies is that UPFs are often considered as a whole, homogenous category, whereas some UPFs are not considered unhealthy.

#### 3.2.2 Allergic diseases

UPF consumption has been associated with an increased prevalence of non-communicable diseases, including allergic conditions, which are among the most common chronic disorders in children worldwide (35, 36). Allergic diseases arise from complex interactions between genetic susceptibility and environmental exposures. A systematic review by Berni Canani et al. highlighted the potential role of UPFs in the development of atopy, asthma, allergic rhinitis, atopic dermatitis, and food allergies, implicating high intakes of fructose, free sugars, and soft drinks, as well as additives such as emulsifiers and artificial sweeteners, in immune dysregulation via alterations of the gut microbiome (37).

UPF-related microbiota changes include increases in potentially harmful bacteria (e.g., Prevotella, Sutterella, Alloprevotella) and reductions in beneficial taxa (e.g., Bacteroidetes, *Faecalibacterium prausnitzii*, Ruminococcus) (38–41). Emulsifiers such as carboxymethylcellulose can disrupt the intestinal epithelial barrier, increasing permeability and allergen sensitization (42).

Epidemiological studies support these mechanisms. Yu et al. reported a 2.5-fold higher risk of allergic sensitization among children who consumed fruit drinks at least 5 times per week compared with those who consumed them less frequently (42). Other studies have linked high carbohydrate intake to asthma severity (43), while the ISAAC study demonstrated associations between frequent fast-food consumption and asthma, rhinoconjunctivitis, and eczema in children and adolescents (44). Together, these findings underscore the role of UPFs in the rising burden of pediatric allergic diseases.

#### 3.2.3 Dental diseases

Diet-related chronic diseases also include dental conditions, particularly dental caries. A meta-analysis by Cascaes et al. reported a 71% increase in the risk of dental caries among children and adolescents with high UPF consumption (45). Sugars—abundant in most UPFs—play a central role in the development of caries (46, 47). Fermentable carbohydrates are metabolized by oral bacteria into lactic acid, lowering salivary pH and promoting enamel demineralization (48). The risk is further amplified by sticky foods and sugar-sweetened beverages, which prolong tooth exposure to fermentable substrates (49).

### 3.3 Maternal UPF consumption during pregnancy and offspring health

Pregnancy involves profound metabolic and physiological adaptations, making maternal diet quality critical for fetal development. Exposure to UPFs may begin before birth, as many women consume these products before conception and throughout pregnancy and lactation (12, 50). Diets high in UPFs are often deficient in long-chain polyunsaturated fatty acids (LCPUFAs), including EPA and DHA, which are essential for neurodevelopment (12, 50). Evidence linking prenatal UPF exposure to offspring outcomes is summarized in Table 3.

**Table 3:** Potential outcomes of maternal exposure to UPFs during pregnancy.

Level	Mechanism/process	Examples of nutritional or chemical factors	Physiological effect on mother or fetus	Potential perinatal/long-term outcomes	References
Nutritional deficiency	Insufficient supply of key nutrients essential for fetal growth and brain development	↓ LC-PUFAs (DHA, EPA), ↓ choline, ↓ iron, ↓ zinc, ↓ high-quality protein, ↓ folate	Impaired neurogenesis, myelination, and neurotransmis-sion; placental dysfunction	↓ Birth weight, delayed cognitive and psychomotor development	(86, 87)
Metabolic dysregulation	Excessive intake of refined sugars and saturated/trans fats leading to insulin resistance and lipotoxicity	High glycemic load, palm oil, hydrogenated fats	Maternal hyperglycemia, oxidative stress, low-grade inflammation	↑ Fetal adiposity, macrosomia, altered glucose metabolism in offspring	(88, 89)
Inflammation and oxidative stress	Diet-induced activation of pro-inflammatory cytokines and reactive oxygen species	Advanced glycation end-products (AGEs), lipid peroxidation products	Placental inflammation, endothelial dysfunction	↑ Risk of preterm birth, neuroinflammation, impaired synaptic plasticity	(90)
Endocrine and epigenetic modulation	Disruption of hormonal signaling and epigenetic programming	Bisphenols, phthalates, artificial sweeteners, emulsifiers	Altered DNA methylation, glucocorticoid receptor dysregulation	Long-term metabolic and behavioral alterations	(91)
Microbiota alterations	Reduced microbial diversity and altered short-chain fatty acid production	Low fiber, high emulsifier content	Increased intestinal permeability, systemic inflammation	Immune dysregulation, neurodevelopmental disturbances	(91)
Direct neurotoxic exposure	Additives or nanoparticles crossing the placental or blood–brain barrier	Titanium dioxide (E171), aluminum nanoparticles, synthetic dyes	Neuronal oxidative stress, microglial activation	Cognitive impairment, emotional dysregulation	(54, 92)

While associations with birth outcomes remain inconsistent (51–53), increasing evidence links maternal UPF intake to impaired neurodevelopment (12, 54, 55). Critical neurodevelopmental processes occur from mid-gestation through early infancy, rendering the fetal brain particularly vulnerable to nutritional insults (54). Excessive intake of UPFs, sugar-sweetened beverages, saturated fats, additives, and nanoparticles may promote oxidative stress, neuroinflammation, and altered synaptic development (12, 54, 56, 57).

Maternal UPF intake may also affect breast milk composition. A randomized crossover trial demonstrated that replacing beef with plant-based UPFs altered milk fatty acid profiles, reducing LCPUFAs and increasing saturated fats derived from tropical oils, with potential implications for infant immune and neurodevelopment (58).

Finally, maternal UPF intake during pregnancy has been associated with an increased risk of atopic dermatitis in infancy, as shown in the MOCEH cohort study (11). While earlier studies focused on overall maternal diet quality (59, 60), these findings highlight the need for further research on UPFs specifically.

### 3.4 UPFs, Mediterranean diet, and plant-based dietary patterns

The Mediterranean diet (MedDiet), recognized by UNESCO as Intangible Cultural Heritage, emphasizes plant-based foods, limited meat intake, and moderate fish consumption (61). Despite its well-established health benefits, adherence to the MedDiet is declining, even in Mediterranean countries, alongside rising UPF consumption (14, 62). The SENDO study documented an inverse relationship between MedDiet adherence and UPF intake (63), with a marked decline in adherence among Spanish children over the past decade (64, 65).

Clinical evidence supports the protective role of the MedDiet against pediatric non-communicable diseases, including non-alcoholic fatty liver disease (NAFLD) (66–68, 93). Conversely, the growing adoption of plant-based diets does not necessarily equate to lower UPF intake. Evidence from the UK suggests that vegetarians may consume a higher proportion of UPFs than meat-eaters (69), highlighting the importance of prioritizing minimally processed plant-based foods.

## 4 Discussion

The increasing consumption of UPFs represents a major public health concern for pediatric populations, posing a substantial threat to the establishment of healthy dietary behaviors from early life. Evidence consistently indicates that children and adolescents—particularly in high-income countries—derive approximately 50–60% of their total daily energy intake from UPFs, largely through frequent consumption of sugar-sweetened snacks, beverages, and industrially prepared meals (14, 33, 70, 71). Importantly, early and sustained exposure to these products is shaped by modifiable familial, socioeconomic, and behavioral determinants. Prolonged breastfeeding, higher parental nutrition literacy, and regular shared family meals are associated with lower UPF intake, whereas excessive screen time, larger household size, and early introduction of complementary feeding are associated to higher intake (14, 19–24). These findings emphasize the pivotal role of the home food environment and support family-centered prevention strategies.

From a clinical and biological perspective, the adverse health effects of UPFs are plausibly explained by their nutritional and structural characteristics. UPFs are typically energy-dense, low in fiber, and engineered to be hyperpalatable, thereby impairing satiety regulation and promoting passive overconsumption (27, 32). As a result, high UPF intake has been associated with excess weight gain, metabolic syndrome, and early cardiometabolic risk factors in pediatric populations, with effects that may persist in adulthood (26, 28, 29, 33). Diets rich in UPFs are also characterized by excessive intakes of saturated fats, free sugars, and sodium, together with reduced micronutrient density, contributing to insulin resistance, dyslipidemia, and hypertension (27, 28, 34). Although physical activity remains a cornerstone of pediatric health, excessive UPF consumption may attenuate its protective effects by sustaining a positive energy balance and unfavorable metabolic profiles (34).

Studies conducted in adult subjects indicate that some UPFs can be healthy; this is why the American Medical Association policy calls for understanding the difference between healthful and unhealthy processed foods. Nevertheless, it is evident that some foods classified as “ultra-processed” are far healthier than others, even if the Nova method for classifying food processing levels is widely used. In a recent study, ultra-processed cereals were associated with a 22% lower risk of incident diabetes in a 2023 analysis of three Harvard cohorts; packaged sweet snacks and fruit-based goods were associated with 13 and 18% lower risks, respectively. On the other hand, ready-to-eat/heat-mixed foods and animal-based items were associated with a 42 and 44% higher incidence of diabetes, respectively. The Harvard researchers noted a “favorable relationship of plant-rich diets compared with animal-rich diets with T2D,” acknowledging this contradiction (72). In another study, savory snacks and animal-based products were associated with increased risk, whereas plant-based alternatives were associated with reduced risk (73). Therefore, UPFs should not be considered as a homogeneous category in the context of plant-based diets in adult subjects (74).

Indeed, animal-based UPFs (such as processed meats) are strongly linked to disease risk, unlike breakfast cereals; in fact, the FDA specifically selected breakfast cereals as a vehicle for folic acid supplementation, which decreased the incidence of spina bifida by 23%. So, it would be incorrect to ban these foods. Likewise, a plant-based burger may be referred to as a “UPF,” but these products are associated with a lower disease risk; therefore, parents may correctly include plant-based burgers rather than meat-burgers in their families’ diet. Mendoza et al. conducted research to evaluate the relationship between overall and group-specific UPF consumption and the incidence of cardiovascular disease (CVD), coronary heart disease (CHD), and stroke in three large prospective cohorts of adults in the United States. Higher total UPF intake was negatively associated with the risk of CVD and CHD among U.S. adults, supported by similar prospective studies from various countries, which also indicated a slightly increased risk for stroke. In a sub-analysis, they found that when sodas and animal-based UPFs were removed from the analysis, the remaining UPF categories showed no association with CVD risk. In conclusion, nutritional recommendations for cardiovascular health should consider the different impacts of specific categories of UPFs (75).

This topic will definitely be worth further research, especially in pediatric subjects, so as not to “ban” all UPFs as unhealthy and to adopt a more tailored, personalized approach.

Beyond metabolic consequences, growing evidence links UPF consumption to immune-mediated and oral diseases. Several studies have associated high UPF intake with allergic conditions such as atopic dermatitis, asthma, and rhinoconjunctivitis (37, 42–44). These associations are biologically plausible, as UPF components—including emulsifiers, artificial sweeteners, and low fiber content—may alter gut microbiota composition, disrupt intestinal barrier integrity, and promote immune dysregulation (37–42). In parallel, oral health is negatively affected by the high concentration of free sugars and fermentable carbohydrates typical of UPFs, which increase the risk of dental caries through acidogenic bacterial metabolism and prolonged tooth exposure (45–49). Together, these findings indicate that the health impact of UPFs in childhood extends beyond weight-related outcomes and involves multiple physiological systems during critical developmental stages.

Maternal consumption of UPFs during pregnancy represents an additional and particularly sensitive exposure window. Prenatal UPF intake has been associated with adverse offspring outcomes, including impaired neurodevelopment, increased adiposity, and a higher risk of immune-mediated disorders (11, 12, 54, 55). Although evidence regarding birth outcomes remains inconsistent (51–53, 76, 77), mechanistic pathways—such as deficiencies in essential nutrients (e.g., DHA, choline, iron), increased oxidative stress, neuroinflammation, and exposure to food additives—provide biological plausibility for long-term effects on fetal programming (12, 50, 54, 56, 57). Moreover, maternal UPF intake during lactation may alter breast milk composition, potentially influencing infant immune and neurodevelopmental trajectories (58). These findings highlight pregnancy and early postnatal life as key windows for preventive nutritional interventions (94–99).

In parallel, the erosion of traditional dietary patterns—particularly the Mediterranean diet—is occurring even in regions where these patterns originated, alongside a rapid increase in the availability and consumption of UPFs (14, 62–65). Evidence from pediatric cohorts shows an inverse relationship between adherence to the Mediterranean diet and UPF intake, with lower adherence associated with poorer metabolic outcomes, including non-alcoholic fatty liver disease (63–66). At the same time, the growing adoption of plant-based dietary patterns does not necessarily translate into lower UPF consumption. Commercially available plant-based alternatives are often highly processed, and studies suggest that vegetarian diets may include equal or even higher proportions of UPFs compared with omnivorous diets (69). These findings underscore the importance of emphasizing food processing level rather than dietary labels alone (100).

This narrative review has several strengths. First, it adopts a life-course perspective, integrating evidence from pregnancy through childhood and adolescence, thereby highlighting critical windows of vulnerability and opportunities for early prevention (7, 8). Second, it synthesizes evidence across multiple outcome domains—including metabolic health, allergic diseases, oral health, and neurodevelopment—providing a comprehensive overview of the pediatric health implications of UPF consumption. Third, the focus on modifiable familial and behavioral determinants allows identification of actionable targets for clinical practice and public health policy (14, 19–22).

Nevertheless, several limitations should be acknowledged. As a selective narrative review, this work does not provide a quantitative synthesis of effect sizes and may not capture all available evidence. Considerable heterogeneity exists among studies with respect to age groups, dietary assessment methods, definitions and quantification of UPF exposure, and outcome measures, limiting direct comparability across findings (14, 26, 33). Most available evidence is observational, precluding firm causal inferences and leaving open the possibility of residual confounding related to socioeconomic status, overall dietary quality, and lifestyle behaviors (27, 28). In addition, dietary intake data are often self- or proxy-reported, which may introduce recall and reporting bias, particularly in pediatric populations. Finally, although mechanistic hypotheses are increasingly supported by experimental and translational research, biological pathways linking UPF consumption to specific pediatric outcomes require further clarification through longitudinal and interventional studies.

Overall, the current body of evidence supports the prioritization of life-course–oriented prevention strategies aimed at reducing UPF consumption, strengthening family-based nutrition education, and promoting minimally processed dietary patterns from pregnancy through childhood and adolescence, thereby reducing the long-term burden of non-communicable diseases (1, 14, 33).

## 5 Conclusion

Taken together, the evidence reviewed underscores the urgent need for comprehensive, life-course–oriented prevention strategies aimed at reducing UPF consumption. Such strategies should prioritize the promotion of unprocessed and minimally processed foods, strengthen family-based nutrition education, and support the development of healthy eating behaviors beginning in pregnancy and continuing through infancy, childhood, and adolescence. Early and sustained interventions during these sensitive developmental periods have the potential to optimize growth, neurocognitive development, immune function, and metabolic health, thereby reducing the long-term burden of non-communicable diseases.

Future research should address several critical gaps. Longitudinal studies with standardized definitions of UPF exposure are needed to clarify causal relationships and identify sensitive windows of vulnerability across the life course. Greater attention should be devoted to prenatal and early postnatal exposure, including the impact of maternal UPF consumption on fetal programming, neurodevelopment, immune maturation, and cardiometabolic risk. Well-designed intervention studies are also required to determine the effectiveness of strategies aimed at reducing UPF intake in families and pediatric populations, particularly those integrating nutritional education, food environment modification, and policy-level actions. In addition, mechanistic research should further elucidate the biological pathways linking UPF consumption to adverse health outcomes, including alterations in gut microbiota, immune regulation, endocrine signaling, and neurodevelopment. Finally, future investigations should explore the health implications of emerging dietary trends, such as highly processed plant-based alternatives, to distinguish nutritionally adequate diets from those that are merely plant-derived but still ultra-processed. Addressing these research priorities will be essential to inform evidence-based dietary guidelines and public health policies that effectively protect child health across generations.
